# Severe leptospirosis after rat bite: A case report

**DOI:** 10.1371/journal.pntd.0008257

**Published:** 2020-07-09

**Authors:** Thais Faggion Vinholo, Guilherme S. Ribeiro, Nanci F. Silva, Jaqueline Cruz, Mitermayer G. Reis, Albert I. Ko, Federico Costa

**Affiliations:** 1 Department of Epidemiology of Microbial Disease, Yale School of Public Health, New Haven, Connecticut, United States; 2 Instituto Gonçalo Moniz, Fundação Oswaldo Cruz, Ministério da Saúde, Salvador, Brazil; 3 Faculdade de Medicina, Universidade Federal da Bahia, Salvador, Brazil; 4 Escola Bahiana de Medicina e Saúde Pública, Salvador, Brazil; 5 Instituto de Saúde Coletiva, Universidade Federal da Bahia, Salvador, Brazil; 6 Institute of Integrative Biology, University of Liverpool, Liverpool, United Kingdom; University of Connecticut Health Center, UNITED STATES

## Presentation of case

**Fig 1:**
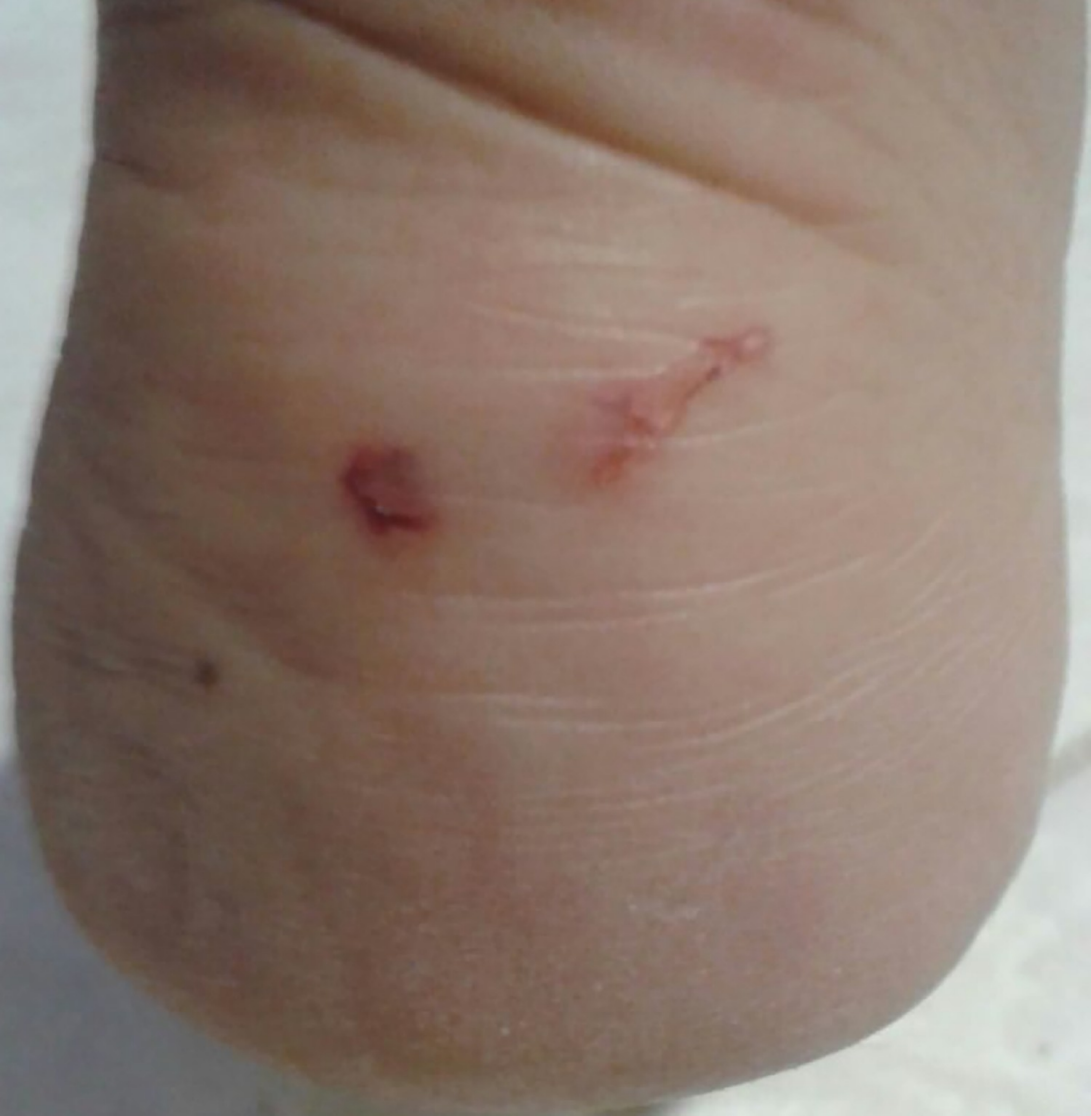
Picture of the rat bite on the right ankle of the patient.

**Fig 2:**
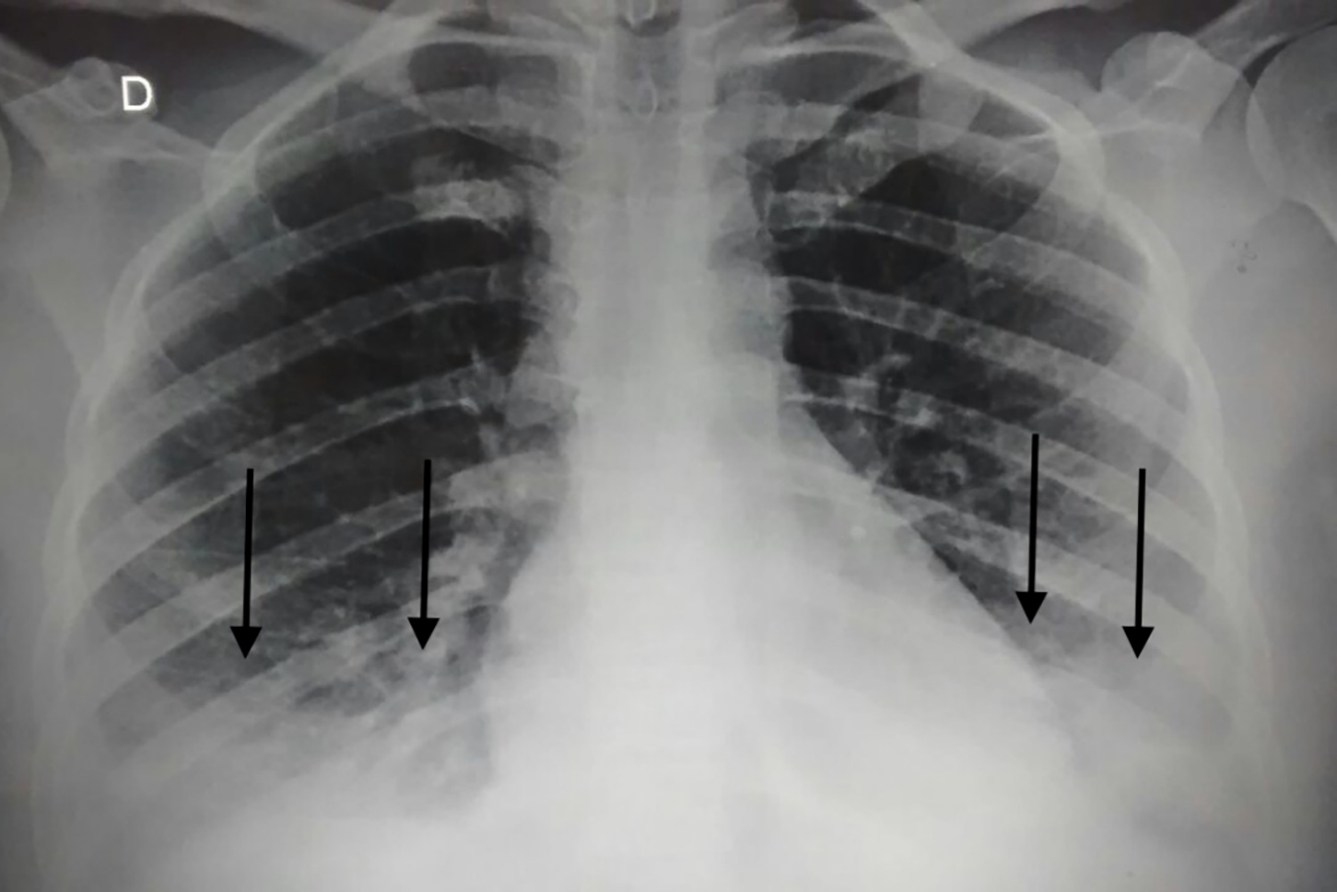
Antero-posterior chest X-ray of the patient during hospitalization, revealing bilateral basilar consolidation, arrows.

A 43-year-old woman presented to an emergency department in Salvador, Brazil, with a two-day history of fever (39.5^o^ to 40.0°C), chills, headache, arthralgia, myalgia, and loss of appetite in the setting of a recent rat bite. She had no previous relevant medical history but reported a street-rat bite on her right ankle 13 days prior to presentation (Fig 1). The rat bite occurred while she was walking to a drugstore in the early evening in December 2014 in a medium-income neighborhood of Salvador, a coastal city in the northeast of Brazil. Shortly after the incident, she went to an urgent care unit where she received tetanus and rabies vaccines and wound care. She denied exposure to other potentially leptospires-contaminated environments, such as water or mud. When the symptoms began, she was seen at the hospital where she received medical examination and laboratory evaluation. Her complete blood count (Day 1) showed discrete anemia, leukocytosis with neutrophilia, and thrombocytopenia (Table 1). Her urinalysis showed hematuria. The erythrocyte sedimentation rate was 24 mm^3^/hr, creatine phosphokinase was 1,182 U/L, and no other pertinent findings were reported. Blood culture showed no growth, and a rapid dengue test was nonreactive. She received intravenous fluids, muscle relaxants, and analgesics and was discharged without a clear diagnosis. Persistent symptoms brought the patient back to the hospital the next day (Day 2) complaining of shortness of breath, diffused myalgia, arthralgia, odynophagia, dry mouth, and hemoptysis as well as cutaneous rashes. Clinical examination recorded a temperature of 38.0°C, blood pressure of 117/72 mmHg, heart rate of 100 beats per minute, respiratory rate of 16 breaths per minute, and oxygen saturation of 99% breathing room air, in addition to dehydration. She was admitted to the hospital that same day—antibiotics (ceftriaxone) and supportive measures were initiated. On Day-3, she developed shortness of breath and crepitus on thorax auscultation at the base of her right lung and had 82% of oxygen saturation at room air. The patient was admitted to the intensive care unit (ICU) for noninvasive respiratory support. Thorax computed tomography and X-ray revealed bilateral diffused consolidation with air bronchogram and a posterior basal laminar stroke of her left lung (Fig 2). The antibiotics were changed to moxifloxacin and cefepime. Oseltamivir and corticosteroids were introduced. Additionally, during Day 3 in the ICU, the patient presented with hemoptysis and pulmonary congestion, likely associated to hypervolemia, which were resolved by the Day 4. The patient was discharged on Day 6 with complete resolution of fever and respiratory symptoms. A definitive diagnosis of leptospirosis was made based on the epidemiological history of rat bite, compatible clinical symptoms, and laboratory tests. A positive immunoglobulin M (IgM) ELISA (Bio-Manguinhos, Rio de Janeiro, Brazil), a positive rapid test for leptospirosis (Bio-Manguinhos), and microagglutination test titers of 1:3,200 directed against *Leptospira interrogans* serovar Copenhageni, in convalescent phase sera sample fulfilled previously described laboratory diagnostic criteria (an acute phase sera was not available for diagnostic testing) [1,2].

**Table 1:** Results of the laboratory tests obtained while the patient was hospitalized.

Laboratory Test	Day 1 (01/12)	Day 3 (01/14)	Day 4 (01/15)	Day 5 (01/16)	Reference Values
Hemoglobin (g/dL)	12.8	11.4	10.6	10	12.0 to 19.0
Hematocrit (%)			31	28	38 to 53
Albumin (g/dL)			2.5		3.5 to 5.5
White blood cell (mil/m^3^)	11.6	84	4.44	11.68	4 to 10 mil/m^3^
Platelets (mil/mm^3^)	110	117	58	83	
Erythrocyte sedimentation rate (mm^3^/h)	24		94		Women: 0–25 / Men: 0–15
C-reactive protein (mg/dL)	17.6			18.3	< 1.0
Prothrombin time (seconds)		14.4		16	14.8
PTT (seconds)	28.7				
INR	0.97				
CPK (U/L)	1,182	1,254	419	650	Women: 30 to 135 / Men: 55 to 170
ALT (U/L)	14	52	62	62	Women: 9 to 52 / Men: 21 to 72
AST (U/L)	24	97	113	86	Women: 14 to 36 / Men: 17 to 59
Troponin I			0.329		< 0.034 ng/mL
Conjugated hyperbilirubinemia					
Gamma-glutamyltransferase					
Creatine (mg/dL)	0.9	1.2		1	Women: 0.5 to 1 / Men: 0.7 to 1.3
Urea (mg/dL)		24	14	26	Women: 15 to 36 / Men: 19 to 43
**Arterial Blood gases**					
** **pH				7.41	
** **pCO_2_ mmHg				34	
** **pO_2_ mmHg				188	
** **SO_2_%				99.7	

ALT, alanine transaminase; AST, aspartate transaminase; CPK, creatine phosphokinase

## Case discussion

Leptospirosis, a zoonotic disease caused by a spirochete of the genus *Leptospira*, is endemic in tropical countries. The incidence of leptospirosis in high-risk areas of Salvador is 20 cases per 100,000 people [3]. The most common mode of transmission is through human exposure on abraded skin or intact mucous membrane to contaminated environments, such as contaminated rat urine or contaminated water or soil [4].

Leptospirosis is characterized by its variable manifestation, and it can be fatal. Its presentation may range from asymptomatic to vital organs involvement. It can lead to acute renal failure, multiple organ disfunction, acute respiratory distress syndrome, and Weil’s disease [5]. Its significant morbidity and mortality highlights the importance of recognizing and identifying how leptospirosis can be acquired[6].

### The presenting case

In the case at hand, we suggest an unusual mode of leptospirosis transmission—direct infection from a rat bite. In tropical countries, individuals exposed to unsanitary conditions typical of urban slums are at greater risk of contracting leptospirosis [1,5,7]. The subject in this case lives in an urban city where leptospirosis outbreaks annually, affecting the population living in areas of poor sanitation infrastructure during the rainy season [8]. However, the subject’s residence is located in a middle-income neighborhood with appropriated sanitary conditions, and she is not in a high-risk occupational group[7]. These findings, together with the temporal association between the rat bite and the development of symptoms support the hypothesis of direct rat-bite transmission causing the disease, rather than the possibility of indirect transmission via exposure to soil or water contaminated with pathogenic *Leptospira*.

Although rat-bite is not a common mode of transmission, a number of cases have been documented [9–13]. As in the case under analysis, previous reports describe cases where leptospirosis was not an immediate consideration given its unspecific symptoms and uncommon mode of transmission. This is the first case reported in South America and serves as an alert and reminder to physicians and public health officials in tropical countries where leptospires are abundant.

Saliva has not been reported as the customary infectious bodily fluid that carries leptospires. When the saliva of a group of wild urine positive rats (*n* = 81) was tested for the presence of *Leptospira* spp., only one sample was found positive [14]. The transmission during a rat bite could be due to short-term saliva contamination during urogenital area grooming [15]. Alternatively, broken skin, a predominant route to cause infection in environments of low exposure [16], could have been a portal entry for leptospires. However, the subject did not report contact with water or soil potentially contaminated with *Leptospira* during the 11 days before symptoms. Lastly, we cannot exclude the hypothesis that the entry for leptospires could have happened through direct contact from residual urine tracked by the rat once the skin was broken.

The lack of pathognomonic presentation for leptospirosis makes a diagnosis dependent on serologic tests. However, if physicians are not associating rat bites with leptospirosis, especially in areas where cases are uncommon, accurate diagnosis will be delayed and the disease will advance. It’s been shown that patients with severe pulmonary hemorrhagic syndrome have a 74% case-fatality ratio [17] and, when admitted to the ICU in the setting of leptospirosis, have a 52% mortality risk [5]. This study aims to raise awareness of leptospirosis in the setting of rat bite in order to avoid inaccurate diagnosis and delay of treatment.

**Key learning points**

- Rat bites may be common occurrence in populations living in environmental settings of high Norway rat abundances, such as slum dwellers.
- Leptospirosis should be part of the differential diagnosis in the setting of a rat bite.
- When considering leptospirosis, health care providers should act promptly in order to avoid development of severe clinical manifestations, such as pulmonary hemorrhagic syndrome.
